# Liver retraction using *n*-butyl-2-cyanoacrylate glue during laparoscopic cholecystectomy

**DOI:** 10.1097/MD.0000000000025879

**Published:** 2021-05-21

**Authors:** Gang Du, Du Kong, Binyao Shi, Zhengchen Jiang, Muguo Aniu, Jinhuan Yang, Hao Zhang, Li Gao, Bin Jin

**Affiliations:** Department of General Surgery, Qilu Hospital of Shan Dong University, Jinan, China.

**Keywords:** laparoscopic cholecystectomy, liver retraction, n-butyl-2-cyanoacrylate glue, surgery

## Abstract

There might be a thick “protrusion” in the visceral surface of hepatic quadrate lobe during the laparoscopic cholecystectomy (LC), which affects the surgical fields and consequently triggers high risks of biliary tract injury. Although n-butyl-2-cyanoacrylate (NBCA) glue has been applied to laparoscopic upper abdominal surgery for liver retraction, there is still no consensus on its safety and feasibility in LC. In this study, we investigated the safety, feasibility, and effectiveness of liver retraction using NBCA glue for these patients which have the thick “protrusion” on the square leaf surface of the liver during LC.

Fifty-seven patients presenting thick “protrusion” hepatic quadrate lobe were included in our retrospective study. We performed LC in the presence of NBCA glue (n = 30, NBCA group) and absence of NBCA glue (n = 27, non-NBCA group), respectively. NBCA was used to fix the thick “protrusion” of the liver leaves to the hepatic viscera surface, which contributed to the revelation of the gallbladder triangle. The operation time, blood loss, postoperative hospitalization, and liver function were compared between the 2 groups.

Both the groups’ patients accomplished the operation in the laparoscopy. There was no mortality and no additional incision during operation. No severe complications including bile duct injury were available after surgery and no postoperative NBCA-related complications occurred after 9- to 30 months’ follow-up. The time of operation in NBCA group showed significant decrease compared with that of non-NBCA group (48.33 ± 16.15 vs 65.00 ± 22.15 minutes, *P* < .01). There were no significant differences in blood loss, postoperative hospital stays, and the preoperative and postoperative liver function between the two groups (*P* > .05). Besides, no significant differences were noticed in major clinical characteristics between the 2 groups (*P* > .05).

Liver retraction using NBCA during LC for thick “protrusion” hepatic quadrate lobe patients is safe, effective, and feasible.

## 1 Introduction

Laparoscopic cholecystectomy (LC), initially reported by Philippe Mouret in 1987, has now been widely promoted in various countries. To date, the procedures can be accomplished by sophisticated and qualified surgeons, but there are still some problems for it. On some occasions, there is a thick “protrusion” on the quadrate lobe of liver, which affects the visualization of surgical field and increases the difficulty of gallbladder trigonometry, as well as increased possibility of biliary tract injury.

In clinical settings, it is essential to establish a clear surgical field and generate adequate working space for safe laparoscopic surgery.^[1]^ To our best knowledge, attenuating the “protrusion” of the quadrate lobe of liver is crucial for adequate working space and generation of a clear surgical field. In the past decades, electrocoagulation hook of the right hand had been commonly utilized to block the “protrusion” of the quadrate lobe of liver. Nevertheless, this method is not conducive to the flexibility of the right hand, disclosure, and operation. Nowadays, several techniques are available for liver retraction in the left and the entire liver. However, the procedures to avoid an additional incision for a retractor are time-consuming, which require intracorporeal suturing or under the assistance of special devices.^[2–5]^ Indeed, these methods are not suitable for the thick “protrusion” retraction of liver leaf surface. In our experiences, we proposed the use of n-butyl-2-cyanoacrylate glue (NBCA) to fix the thick “protrusion” of the liver leaf surface to the liver surface during LC.^[6]^ The procedure contributes to the visualization of the gallbladder triangle. In this study, we aim to investigate the safety and effectiveness of NBCA as a means of fixing a thick “protrusion” on the square leaf surface of liver in LC.

## 2 Methods

### 2.1 Patients

In our retrospective study, 57 LC patients presenting thick “protrusion” hepatic quadrate lobe were divided into NBCA group (n = 30) and non-NBCA group (n = 27). The clinical data of the 2 groups’ patients were collected during January 2016 to September 2019 in Qilu Hospital, including age, sex, weight, operation time, blood loss, postoperative hospital stay, and the preoperative and postoperative liver function. In the NBCA group, the NBCA glue was utilized to completely expose the surgical vision. Meanwhile, in the non-NBCA group, electrocoagulation hook of the right hand was utilized to block the “protrusion” of the quadrate lobe. All cases signed the informed consent. The study protocols were approved by the institutional review committee.

### 2.2 Operative technique

Traditional surgery was performed to the cases with no thick “protrusion” on the visceral surface of quadrate lobe of liver during the LC. For the cases with a thick “protrusion” of the liver square lobe visceral surface under the LC, the NBCA method was utilized. Traditional laparoscopic surgery was performed by qualified surgeons. All patients adopt the general anesthesia under a supine position. The 3-trocar method or the 4-trocar method was utilized for the operation. The curved incision under the umbilical with a length of 10 mm served as an observation hole. Then 3 operating holes with a diameter of 10 mm, 5 mm, and 5 mm were made on the cartilago ensiformis, below right costal margin, and the right axillary frontline, respectively.

### 2.3 Technique of retraction

**Figure 1:**
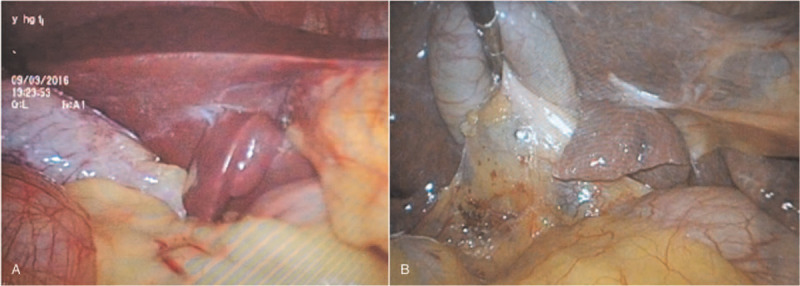
The thick “protrusion” (A, B) of the liver square lobe visceral surface affected the surgical field of vision.

**Figure 2:**
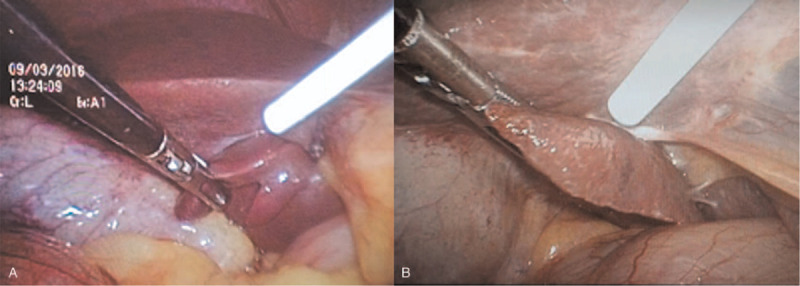
The n-butyl-2-cyanoacrylate glue glue was sprayed on the thick “protrusion” (A, B) of the liver square lobe visceral surface.

On some occasions, some patients may present a thick “protrusion” of visceral surface of quadrate lobe of liver, which interferes with the vision of surgical field (Fig. 1A and B). For these cases, the retraction method using NBCA was performed. First, the glue was infused into the sterile bottle connected to the sprayer and catheter. Then, the left hand was pulled through the trocar (5 mm) to the thick protrusion of the liver side. Afterwards, a catheter in the right hand was inserted into the abdominal cavity through the trocar with a dimension of 10 mm. Secondly, the medical glue (0.5 mL) was sprayed on the thick “protrusion” of the liver side of the liver (Fig. 2A and B). When spraying medical glue, the lens should be withdrawn in order to prevent glue contamination. Third, the thick “protrusion” of the liver square lobe visceral surface was pressed onto the liver visceral surface for about 15 seconds. Finally, the thick “protrusion” on the visceral surface of quadrate lobe of liver was not deliberately released to restore its original position.

For each patient, the liver adhesion time, duration of operation, blood loss, postoperative hospital stays, and complications were recorded. Additionally, parameters used to test the liver function including alanine aminotransferase (ALT), aspartate aminotransferase (AST), γ-glutamyl transpeptidase (GGT) and total bilirubin (TBIL) before and after operation were recorded.

### 2.4 Statistical analysis

SPSS 19.0 software (IBM; SPSS Inc, Chicago, IL) was used for the data analysis. All data were expressed as mean ± standard error. The data of age, weight, time of operation, blood loss, postoperative hospital stay, ALT, AST, GGT, and TBIL were analyzed by Student *t* test. The sex data were analyzed by Pearson *χ*^2^ test. *P* < .05 was considered to be statistically significant.

## 3 Results

### 3.1 Advantages of the retraction technique using NBCA

**Figure 3:**
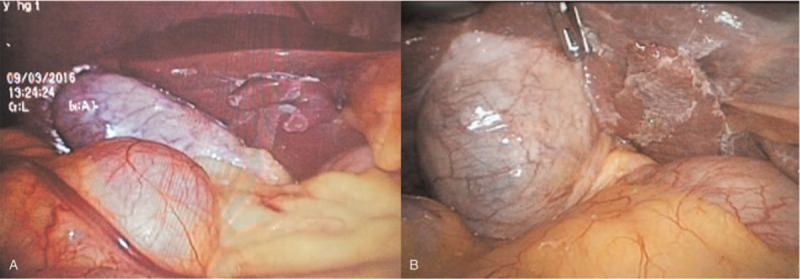
Exposure (A) and surgical visualization (B) obtained after liver adhesion.

This procedure avoided the influence of the large “protrusion” of the liver square lobe visceral surface on the visualization of operation field, and facilitated to the exposure of the gallbladder triangle (Fig. 3A and B). Besides, the right hand of the surgeon was free as there was no need to block the drooping of “protrusion” of the quadrate lobe of liver using the electrocoagulation hook.

### 3.2 Treatment efficiency of NBCA

There were no significant differences in major clinical characteristics between the 2 groups (*P* > .05, Table 1). All the patients in the 2 groups received complete operation under the laparoscopy, and no one received open surgery. There was no mortality and no additional incision during operation. There were no severe complications including bile duct injury after surgery and no postoperative NBCA-related complications in the 9- to 30 months’ follow-up.

**Table 1:** Clinical characteristic of the patients.

Variable	NBCA group (n = 30)	Non-NBCA group (n = 27)	*P*
Age, y	52.60 ± 15.09	52.51 ± 13.77	.983
Sex			1.000
Male	17	15	
Female	13	12	
Weight, kg	65.17 ± 8.27	63.70 ± 12.54	.610
Time of operation, min	48.33 ± 16.15	65.00 ± 22.15	.002
Blood loss, mL	25.17 ± 11.33	28.52 ± 14.66	.336
Postoperative hospital stays, day	3.77 ± 1.17	3.81 ± 1.21	.879

The time of operation in NBCA group showed significant decrease compared with that of non-NBCA group (48.33 ± 16.15 vs 65.00 ± 22.15 minutes, *P* < .01). The average time of liver adhesion was 0.5 minutes. There were no significant differences in blood loss (25.17 ± 11.33 vs 28.52 ± 14.66 minutes, *P* > .05), postoperative hospital stays (3.77 ± 1.17 vs 3.81 ± 1.21 minutes, *P* > .05), and the preoperative and postoperative liver function between the 2 groups (*P* > .05, Table 2).

**Table 2:** Perioperative parameters in 2 groups.

Variable	NBCA group (n = 30)	Non-NBCA group (n = 27)	*P*
ALT, U/L			
Preoperative	26.27 ± 24.04	23.00 ± 19.34	.577
Postoperative	35.50 ± 24.15	36.48 ± 18.75	.866
AST, U/L			
Preoperative	24.83 ± 18.95	22.30 ± 15.51	.488
Postoperative	39.73 ± 24.87	39.93 ± 15.25	.972
GGT, U/L			
Preoperative	53.07 ± 74.95	55.41 ± 82.20	.911
Postoperative	54.80 ± 77.04	55.19 ± 54.03	.983
TBIL, μmol/L			
Preoperative	14.83 ± 9.01	14.25 ± 6.17	.781
Postoperative	17.98 ± 7.76	19.35 ± 6.63	.478

The operation time of liver adhesion showed significant decreased. Compared with these received conventional surgery, no significant differences were noticed in liver function tests after operation. Besides, the liver retraction resulted in no deterioration of liver function damage. During the operation, the protrusion of the square lobe of the liver adhered to the liver viscera continuously. There were no complications related to liver retraction during the operation. All patients were followed up 9- to 30 months’ after procedures. There were no complications related to liver retraction technology (Table 2).

## 4 Discussion

LC has been well mastered by qualified liver and gallbladder surgeons in different countries.^[7–10]^ However, in some cases underwent LC, there might be a thick “protrusion” of liver leaves.^[11,12]^ It often covers the gallbladder triangle, which then affects the visualization of surgical field. Additionally, it may induce a high risk of biliary tract damage. In the past decades, right-handed electrocoagulation hook was utilized for the surgery, in which the electrocoagulation hook was used to pick up the pendulous thick “protrusion” on the visceral surface of the quadrate lobe of liver in order to expose the gallbladder triangle and visualize the surgical fields. However, such method greatly limits the flexibility of the right hand and is not conducive to surgical operations. In this study, by using NBCA, the pendulous thick “protrusion” on the visceral surface of the quadrate lobe of liver adhere to the surface of the liver square leaves. On this basis, the gallbladder triangle was exposed, and the surgical field was clearer. Meanwhile, the right hand was free during these procedures, and the electrocoagulation hook of the right hand was more flexible for operation.

Various liver retraction techniques have been described for laparoscopic procedures like cholecystectomy and gastrectomy.^[5,13–16]^ In addition, some studies had described satisfactory results with the use of a flexible silicone disk to facilitate liver retraction.^[17–19]^ However, these methods and techniques are generally for the left outer lobe of the liver or the contraction of the entire liver. To date, there are few reports about the retraction technique of “protrusion” of the liver square lobe visceral surface, which sometimes appears in LC. This study confirmed the safety and efficacy of NBCA glue in creating adhesion between the thick “protrusion” of the liver square lobe visceral surface and hepatic visceral surface in LC. Meanwhile, iatrogenic liver injury caused by mechanical tractor was avoided.^[20,21]^

Our technique lasted for about 0.5 minutes, and no special instruments were needed. In clinical practice, we often use NBCA glue as hemostasis for surgical wounds. We use a small part of the NBCA glue to make the “protrusion” of the square lobe of the liver retraction. No additional adhesive was needed, and no additional medical costs were generated. The “protrusion” adhesion was achieved successfully in all patients and lasted until the end of the operations, On the basis that NBCA glue can be degraded completely and absorbed after 9 months,^[22,23]^ we have not attempted to separate the adhesion. There were no complications related to liver retraction during and after operation. Besides, those received liver retraction using NCBA glue did not present biliary duct injury. Moreover, there were no complications in the long-term follow-up. The operation time of NBCA group was shorter than non-NBCA group. The liver retraction involving the application of NBCA glue would not cause additional bleeding. Moreover, no significant differences were noticed in postoperative ALT and AST between the 2 groups, which suggested that liver retraction using NBCA glue during LC may not induce additional liver damages. Our data implied that liver retraction using NBCA glue was a simple, safe, and effective way to enhance exposure in LC for these patients which have the thick “protrusion” on the square leaf surface of the liver. A clear view of surgical field and suitable workspace is very important. In our study, the shortened time in NBCA group may be related to the increase of surgical exposure by using the liver retraction technique. These benefits may produce a clear view of surgical field and suitable workspace. Besides, the assistant may undergo less labor-extensive procedures to retract liver. A good surgical field of vision is essential for LC to prevent bile duct damage and reduce the operation time. The “protrusion” of the square liver lobe is located above the triangle of the gallbladder, blocking the surgical field of vision. The previous method is to use the electrocoagulation hook of the right hand to block the “protrusion” on the visceral surface of the square liver during the operation. These two operation methods must be completed at the same time. This method limits the flexibility of the right hand and is not conducive to the exposure of the visual field and the delicate operation. The use of NBCA glue for liver retraction increases the exposure of the surgical field of view. The “protrusion” of the square lobe of the liver is adhered to the liver. For the occlusion above the gallbladder triangle, it increases the flexibility of the operator's right hand, making it more convenient for the operator to expose the gallbladder triangle during the operation, together with performing delicate operations. This could effectively prevent and reduce bile duct damage, and shorten the operation time.

There were limitations indeed. First, this method is only applicable to patients with the thick “protrusion” on the visceral surface of the quadrate lobe of liver, because only some patients have a thick “protrusion” on the visceral surface of the quadrate lobe of liver. Secondly, the sample size is not large enough. Thirdly, in this study, we cannot design random studies. Fourthly, the follow-up time is very short in this study. On this basis, a longitudinal study with longer follow-up should be required to assess the long-term outcomes in NBCA group.

## 5 Conclusions

In conclusion, patients in NBCA group showed shortened operation time compared with non-NBCA group. Compared with the conventional technique, it causes no additional complications and damages. Our data concluded that liver retraction using NBCA glue during LC was safe, effective, and feasible for these thick “protrusion” square lobe patients.

## Author contributions

DG wrote the manuscript; JB revised the manuscript; KD, SBY, JZC did the data analysis; AMG, YJH, ZH, GL did the data collection.

**Conceptualization:** Du Kong.

**Data curation:** Du Kong.

**Formal analysis:** Du Kong, Binyao Shi, Li Gao.

**Funding acquisition:** Binyao Shi.

**Investigation:** Binyao Shi, Li Gao.

**Methodology:** Zhengchen Jiang.

**Project administration:** Zhengchen Jiang, Jinhuan Yang.

**Resources:** Zhengchen Jiang, Hao Zhang, Li Gao.

**Software:** Zhengchen Jiang, Muguo Aniu, Jinhuan Yang, Hao Zhang.

**Supervision:** Muguo Aniu, Jinhuan Yang.

**Validation:** Muguo Aniu, Hao Zhang.

**Visualization:** Muguo Aniu, Jinhuan Yang, Hao Zhang.

**Writing – original draft:** Gang Du.

**Writing – review & editing:** Bin Jin.
